# Doxazosin GITS (gastrointestinal therapeutic system) is an actor to consider in the control of hypertension. A narrative review

**DOI:** 10.1038/s41440-025-02432-4

**Published:** 2025-11-05

**Authors:** Miguel Camafort, Hae-Young Lee, Hung-Yu Chang, Bancha Satirapoj, Kazuomi Kario

**Affiliations:** 1 Hypertension Unit, Department of Internal Medicine, Hospital Clinic, University of Barcelona, Barcelona, Spain; 2 Biomedical Research Network Center for the Pathophysiology of Obesity and Nutrition (CIBER-OBN), Carlos III Health Institute, Madrid, Spain; 3 “Clínic-Barcelona” Research Foundation, August Pi i Sunyer Biomedical Research Institute (IDIBAPS), Barcelona, Spain; 4 Division of Cardiology, Department of Internal Medicine, Seoul National University Hospital, Seoul, Republic of Korea; 5 Department of Internal Medicine, Seoul National University College of Medicine, Seoul, Korea; 6 Heart Center, Cheng Hsin General Hospital, Taipei, Taiwan; 7 Faculty of Medicine, School of Medicine, National Yang Ming Chiao Tung University, Taipei, Taiwan; 8 Division of Nephrology Department of Medicine, Phramongkutklao hospital and College of Medicine, Bangkok, Thailand; 9 Division of Cardiovascular Medicine, Department of Medicine, Jichi Medical University School of Medicine (JMU), Tochigi, Japan

**Keywords:** Morning hypertension, Implemental hypertension, Digital hypertension

## Abstract

Hypertension remains a leading global cause of cardiovascular morbidity and mortality, yet blood pressure control rates remain suboptimal despite advances in antihypertensive therapy. Multiple barriers—including cost, side effects, therapeutic complexity, and physician inertia—impede effective management, particularly in aging populations and low-resource settings. This review evaluates the therapeutic role of doxazosin, particularly in its extended-release gastrointestinal therapeutic system formulation, as a potential adjunct in contemporary hypertension treatment, including resistant hypertension.

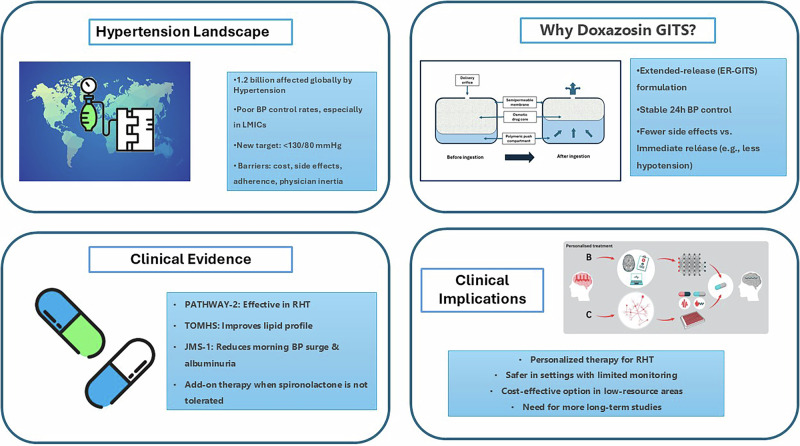

## Introduction

Hypertension (HT) remains one of the leading causes of morbidity and mortality worldwide, affecting over 1.2 billion people as of 2019 [1]. This condition significantly increases the risk of ischemic heart disease, peripheral arterial disease, heart failure, stroke, dementia, and kidney disease, making its control a crucial public health priority. Despite the availability of numerous antihypertensive agents, blood pressure (BP) control remains suboptimal, in 2019, only 23% of women and 18% of men with HT achieved BP control worldwide [1]. Particularly, awareness, treatment, and control rates vary significantly worldwide [2, 3]. Moreover, treatment disparities persist between high- and low-income regions, further complicating HT management [3]. High-income countries such as Canada and South Korea reported control rates exceeding 50% [4], whereas low- and middle-income countries in sub-Saharan Africa and Asia reported rates below 10%. Notably, Studies have shown that Asian patients exhibit a stronger correlation between BP levels and stroke incidence compared to Western populations [5].

Effective BP control is essential for reducing cardiovascular morbidity and mortality. The benefits of lowering BP extend across different baseline BP levels and apply to both primary and secondary prevention of cardiovascular disease [6]. Thus, recent HT guidelines recommend a more aggressive BP target (<130/80 mmHg), demonstrating that tighter BP control leads to improved cardiovascular outcomes [7–9]. In particular, achieving BP control is crucial for special populations, including elderly patients, individuals with diabetes, and those with chronic kidney disease [4, 10, 11]. However, achieving these targets remains challenging due to patient nonadherence, side effects of medications, and physician inertia.

Despite advancements in antihypertensive therapy, BP control rates remain stagnant due to multiple barriers. The cost of medications remains a significant concern, particularly in low- and middle-income countries where access to newer antihypertensive agents is limited. Additionally, adverse effects associated with various drug classes, such as dry cough with angiotensin converting enzyme (ACE) inhibitors, electrolyte disturbances with diuretics, and gynecomastia and sexual disturbance with spironolactone, contribute to reduced adherence. Patients with multiple comorbidities often require complex medication regimens, further complicating BP management. Aging populations also present challenges, as older patients frequently have a higher burden of polypharmacy and increased sensitivity to antihypertensive drugs. Furthermore, physician inertia, including reluctance to intensify treatment despite suboptimal BP control, continues to hinder progress in achieving BP targets. These factors collectively contribute to the persistently low control rates despite the availability of effective antihypertensive therapies.

This highlights the need for alternative therapeutic strategies, including the potential role of α1-adrenergic blockers such as doxazosin. In Asia, calcium channel blockers (CCBs) and renin-angiotensin-aldosterone system (RAAS) inhibitors remain the most frequently prescribed antihypertensive agents [3]. However, studies suggest that combination therapy, particularly involving diuretics or alpha-blockers such as doxazosin in extended-release formulation, may be more effective in achieving optimal BP control [12]. Despite this, alpha-blockers are underutilised, primarily due to concerns regarding side effects such as postural hypotension. Given the significant unmet need in BP management, doxazosin-ER warrants further exploration as a viable adjunctive therapy, especially for patients with resistant HT (RHT). Recent trials have indicated that doxazosin-ER, when used in combination with other antihypertensive agents, can be a potential option for patients struggling to reach BP targets on conventional monotherapy.

## What is resistant hypertension (RHT)?

Many patients experience uncontrolled HT despite pharmacological treatment. To define this condition, medical societies have introduced the term RHT [11]. Although RHT is not a distinct disease entity, it is reported in approximately 10–20% of patients with HT [13, 14]. Compared to individuals with well-controlled blood pressure, those with RHT face a significantly higher risk of target-organ damage and adverse cardiovascular events [11]. Furthermore, patients with RHT are more likely to have an underlying secondary cause—often at least partially reversible—compared to those who achieve adequate BP control [15]. Despite slight variations in definitions across guidelines and consensus statements (Table 1) [11, 16–19].

**Table 1:** Comparison of resistant hypertension (RHT) definition in different publications

Publications /Year	Definition	Suggestion
American Heart Association Scientific Statement, 2018	Above-goal elevated BP in a patient despite the concurrent use of 3 antihypertensive drug classes, commonly including a long-acting CCB, a blocker of the RAS (ACEi or ARB), and a diuretic	Various methods of lifestyle interventions, pharmacological treatments and device-based treatments.
National Institute for Health and Care Excellence Guideline, 2019	If HT is not controlled in adults taking the optimal tolerated doses of an ACEi inhibitor or an ARB plus a CCB and a thiazide-like diuretic	Add a 4th antihypertensive drug as step 4 treatment or consult a specialist.
Japanese Society of Hypertension Guideline, 2019	BP does not decrease to the target level despite the use of three different antihypertensive drugs, including a diuretic	Identify factors contributing to RH and address them accordingly, optimize antihypertensive drug therapy, and consider renal denervation.
Canada’s hypertension guideline, 2020	BP above target despite 3 or more BP-lowering drugs at optimal doses, preferably including a diuretic (and usually a RAS blocker and a CCB)	Refer to providers with expertise in the diagnosis and management of HT.
Thai Hypertension Society Guideline, 2024	BP above goal despite the use of ≥3 antihypertensive agents of different classes, typically including a long-acting CCB, RAS blocker, and a diuretic	Identify and address factors contributing to RH, optimize antihypertensive therapy, and consider renal denervation when appropriate.
European Society of Cardiology Hypertension Guidelines, 2024	Office systolic and diastolic BP values above target, despite a treatment strategy involving lifestyle measures and maximum doses of diuretics, RAS blockers, and CCBs	Consider referral to clinical centers with expertise in HT management for further evaluation and advanced diagnostic testing.

*ACEi* Angiotensin-converting enzyme inhibitor, *AHA* American Heart Association, *ARB* Angiotensin receptor blocker, *BP* Blood pressure, *CCB* Calcium channel blocker, *RAS* Renin-angiotensin system, *RH* RHT

The 2018 American Heart Association (AHA) scientific statement defines RHT as the condition where BP values remain elevated above target despite concurrent use of three antihypertensive agents, including a diuretic. All agents should be administered at maximum or maximally tolerated doses and at the appropriate dosing frequency. The 2023 ESH guidelines define RHT as HT in patients who fail to lower office BP to European Society of Hypertension (ESH) treatment goals (BP levels below <140/90 mmHg) once appropriate lifestyle measures and treatment with optimal or best tolerated doses of three or more drugs have been initiated. Insufficient BP control should be confirmed by out-of-office BP measurements showing uncontrolled 24 h BP values. Evidence of adherence to therapy and exclusion of secondary causes of HT are required to define RHT, otherwise RHT is only apparent and called pseudo-RHT.

The guidelines recommend thoroughly confirming uncontrolled office BP values through measurement of BP values outside of the office, confirming adherence to therapy, and properly ruling out secondary causes of HT when RHT is suspected. Pseudo-RHT is the condition where office BP shows lack of BP control, while ambulatory BP measurement (ABPM) or home BP measurement (HBPM) shows well-controlled BP. Masked RHT is the condition where office BP values are below target, showing good control, but ABPM or HBPM values clearly show lack of BP control. Refractory HT is a type of RHT where BP remains uncontrolled despite being in five or more antihypertensive drug classes, including a diuretic.

The global prevalence of RHT is challenging to determine due to the varying definitions of BP control in guidelines and the presence of pseudo-resistance in population studies. A meta-analysis of 91 studies estimated the global prevalence of RHT but only found a true RHT prevalence of 10.3%. True RHT was found in 22.9% of patients with CKD, 56.0% of renal transplant recipients, and 12.3% of geriatric individuals. In Canada, 5.3% of subjects with HT had apparent RHT, with the majority being female and aged 70 or older. A longitudinal study from the UK (1996–2004) found an increase in RHT incidence among patients with HT from 0.93 per 100 patient-years to 2.07 per 100 patient-years. Selection bias in registries and clinical trials could explain the higher prevalence of apparent RHT. Various guidelines recommend that patients with RHT should be considered for referral to clinical centers with expertise in HT management for further testing [16, 18–20].

Certain medications, such as nonsteroidal anti-inflammatory drugs, sympathomimetics, and hormonal therapies, can elevate BP and attenuate the effectiveness of antihypertensive agents. Moreover, poor adherence to lifestyle modifications (e.g., high-sodium diet, physical inactivity, smoking, excessive alcohol consumption) or antihypertensive therapy should be assessed as potential contributors. Screening for secondary HT is essential. While a detailed discussion of diagnostic tests and evaluation methods is beyond the scope of this review, common causes of secondary HT, including primary aldosteronism, renal parenchymal disease, renovascular disease, obstructive sleep apnea, and endocrine disorders (e.g., pheochromocytoma, Cushing’s syndrome, hypo-/hyperthyroidism)—should be systematically evaluated in clinically suspected cases [21].

## Alpha-blockers in hypertension

Alpha-blockers, also known as α-adrenergic antagonists, are effective in lowering BP by selectively blocking alpha-1 adrenergic receptors on vascular smooth muscle. This blockade inhibits the vasoconstrictive effects of catecholamines, leading to vasodilation and a reduction in systemic blood pressure. Alpha-blockers also influence lower urinary tract symptoms in men with benign prostatic hyperplasia (BPH) by relaxing smooth muscle in the prostate and bladder neck.

While alpha-blockers are not typically considered first-line agents for essential HT management, they retain clinical relevance in specific scenarios, such as managing resistant HT and concomitant HT and BPH. Contemporary HT guidelines generally recommend a stratified approach to antihypertensive therapy, emphasizing the use of first-line agents like thiazide diuretics, ACE inhibitors, ARBs, and CCBs. Alpha-blockers are typically positioned as a secondary or add-on therapy, not being recommended as monotherapy for uncomplicated HT in patients without compelling indications. In patients with resistant hypertension, they may be considered in case of intolerance to spironolactone or as a fourth-line agent and may be used earlier in the treatment algorithm in patients with compelling indications, such as concomitant BPH. In Table 2 the current recommendations from international HT guidelines are shown [8, 9, 17–20, 22–25].

**Table 2:** Alpha-blocker recommendations in hypertension guidelines

Country/region/year/ref	Alpha-blocker role	Typical agents	Notes
USA (AHA 2017) [22]	Not first-line; maybe considered as second line in men with BPH	Doxazosin, Prazosin, Terazosin	Associated with orthostatic hypotension, especially in older adults. ALLHAT trial reported increased risk of heart failure.
Europe (ESC 2024) [9]	Considered for resistant hypertension after failure of first line therapies	Doxazosin	Used after spironolactone and beta-blockers. Also indicated for pheochromocytoma (PPGL).
Europe (ESH 2023) [8]	Third-line agent; appropriate in specific cases (e.g., BPH)	Doxazosin	Caution due to ALLHAT findings but shown to be safe in ASCOT and PATHWAY-2.
UK (NICE 2019) [17]	Step 4: Therapy for resistant hypertension when serum K + > 4.5 mmol/L	Doxazosin	Not a first-line agent; alternative to spironolactone if contraindicated.
Japan (JSH 2019) [18]	Used in patients with BPH, pheochromocytoma, or morning hypertension	Long acting α1-blockers	Initiate with low dose due to risk of orthostatic hypotension. Rare reflex tachycardia.
China (2018 guideline) [23]	Considered in elderly men with BPH	α1-β receptor blockers	Class IIa recommendation. Orthostatic hypotension risk in elderly patients.
Taiwan (2022) [24]	Resistant Hypertension, HT with Prostate Benign Hypertrophy, treating Pheochromocytoma	Doxazosin	Orthostatic hypotension can be managed with increased salt intake.
South Korea (KSH2018) [25]	Not included in standard treatment algorithms	Not specified	No specific recommendation for alpha-blockers in general hypertension management.
Canada (CHEP 2020) [19]	Not recommended as initial therapy	-	Preference given to agents with proven cardiovascular outcome benefits.

## Doxazosin-immediate release (IR) and doxazosin extended release (ER)

**Fig. 1:**
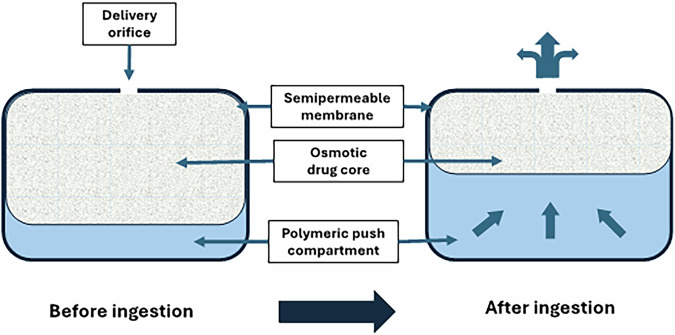
Diagrammatic representation of the gastrointestinal therapeutic system (GITS)

The risk of symptomatic hypotension is one of the adverse effects associated to doxazosin-IR. Usually, a multiple-step titration regimen is advised to mitigate it. The osmotically driven, controlled-release technology employed in the ER-gastrointestinal therapeutic system (GITS) formulation of doxazosin enables a more incremental drug delivery, thereby significantly reducing serum peak-to-trough ratios and the necessity for titration (Fig. 1) [26].

In comparison to doxazosin-IR, clinical pharmacology studies demonstrated that doxazosin-ER-GITS had a higher minimum plasma concentration, a prolonged time to attain maximum plasma concentration, and a lower maximum plasma concentration, leading to a more progressive absorption of doxazosin. The bioavailability of a single doxazosin-ER-GITS tablet was ~75% of that of doxazosin IR. In vitro studies have shown that the release rates of the ER-GITS tablet are not affected by pH in the range of 1.2 (gastric) to 7.5 (intestinal) or by swirling rates that represent gastrointestinal motility. This formulation allows for an initial dose of 4 mg once daily, compared to doxazosin IR, which is initiated at 1 mg/day and titrated, thereafter, to a higher therapeutically effective dose. For both young adult and elderly subjects, and males and females, the pharmacokinetics of 8 mg of doxazosin-ER-GITS once daily for 7 days was comparable to the two 4 mg tablets. Thus, doxazosin-ER-GITS therapy can be initiated at a therapeutic dose of 4 mg with reduced hemodynamic side-effects [27]. The single-dose study assessed the fundamental pharmacokinetic profile and dietary effects after a high-fat meal, while the multiple-dose study evaluated the pharmacokinetic characteristics at constant state.

The ER-GITS formulation of doxazosin provides numerous advantages over the traditional immediate-release (IR) version. In a study evaluating the efficacy and tolerability of three treatment arms, Doxazosin GITS (4 mg or 8 mg once daily), Doxazosin standard (1 mg to 8 mg once daily), and placebo once daily, the authors found that Doxazosin ER GITS is more effective. The study included 392 patients exhibiting mild HT. The ER-GITS system makes it easier for patients to stick to their medication plans by allowing an initial dose of 4 mg once a day without having to adjust the dose. ER-GITS results in a diminished maximum plasma concentration, an extended duration to attain that concentration, and an elevated minimum concentration, consequently mitigating peak-to-trough fluctuations. This is due to improved pharmacokinetics. Clinical trials demonstrate that ER-GITS exhibits a markedly reduced incidence of all-cause adverse events and treatment discontinuations when compared to doxazosin IR. The likelihood of discontinuation due to adverse events was 4.3% for GITS and 11.6% for standard doxazosin, indicating a statistically significant difference (p = 0.049). ER-GITS showed a lower rate of certain side effects.

The main advantages of GITS are as follows:

Discontinuation Rates: The standard formulation had a discontinuation rate of 11.6%, while only 4.3% of patients on ER-GITS stopped treatment because of side effects (*p* = 0.049). These benefits are especially clear in patients younger than 65, where GITS led to a much lower rate of discontinuation due to side effects (1.7% vs. 12.4% for standard, *p* = 0.002).

Reduced Adverse Effects: ER-GITS exhibited lower rates of vertigo (3.7% vs. 6.5%), asthenia (4.3% vs. 5.2%), back pain (1.9% vs. 3.9%), and anxiety (0.6% vs. 2.6%) in comparison to the standard formulation. There were no cases of syncope. ER-GITS, on the other hand, reported no cases of syncope, while the standard formulation had two cases (1.3%).

In summary, doxazosin ER-GITS not only works as well as doxazosin IR but it also makes it easier for patients to take their medicine and stick to their treatment plan by making the dosing process easier and lowering side effects [27, 28].

In an open-label, non-comparative, multicentre trial aimed at evaluating the effects of doxazosin ER-GITS (gastrointestinal therapeutic system) on the 24-hour BP profile in patients with stage 1 to stage 2 primary hypertension, utilising ABPM, 17 patients with stage 1–2 primary HT were recruited. After a two-week washout period, participants received a daily morning dose of 4 mg doxazosin GITS for six weeks, along with 24-hour ABPM both before and after the treatment. The linear analysis revealed significant reductions in daytime, nighttime, and total 24-hour means for systolic BP and diastolic BP from baseline following treatment, while there was no significant change in heart rate (HR). The administration of doxazosin-ER-GITS was well-tolerated, and a single morning dose of 4 mg significantly decreased ambulatory systolic BP and diastolic BP over a 24-hour period while maintaining a normal 24-hour BP and HR rhythm profile [27, 28].

Another study comparing the efficacy and tolerability of doxazosin-ER-GITS in patients with mild HT evaluated its effectiveness and tolerability compared to doxazosin IR and placebo. The primary outcome measure was the proportion of patients who achieved the target BP response. The study found that 92 of 156 patients (59.0%) on doxazosin-ER-GITS and 86 of 152 patients (56.6%) on doxazosin standard achieved a goal BP response 24 h post dose on the final visit, compared to 25 of 70 patients (35.7%) on placebo. The most frequently reported adverse effects were headache, vertigo, and asthenia. Syncope was not reported in the doxazosin-ER-GITS group [26].

## Where could doxazosin GITS ER be a good treatment choice

(1) RHT management in cases of MRA intolerance.

The PATHWAY-2 trial was a double-blind, placebo-controlled crossover trial, conducted in the UK, involving hypertensive patients with RHT. Patients were rotated through four cycles of once daily oral treatment with spironolactone 25–50 mg, doxazosin modified release 4–8 mg, bisoprolol 5–10 mg, and placebo. The study aimed to evaluate the effectiveness of treatment for patients with high BP. Spironolactone showed a reduction of 12.8 mmHg in home systolic BP and of 20.7 mmHg in office systolic BP, while doxazosin-ER-GITS showed a reduction of 8.7 mmHg in home BP and of 16.3 mmHg in office systolic BP, as for bisoprolol reductions were of 8.3 mmHg in home BP and of 16.3 mmHg in office systolic BP. They were no differences in serious or any adverse events between groups [29]. Some of the criticisms of the Pathway-2 trial include the selective reporting of outcomes: The report included only one of the three prespecified primary outcomes, as well as certain secondary and no prespecified outcomes that lacked a clear justification. *Short Follow-up Period: The limited duration of the trial raises concerns regarding the sustainability of the blood pressure-lowering effects of spironolactone and the possibility of unobserved long-term adverse effects. Attrition Bias: A larger dropout rate in the doxazosin group may have introduced bias, which could have impacted the validity of the results. Risks of Discontinuing Medication: The crossover design, which necessitated an abrupt transition between medications, could present a safety hazard. Unreported Drug Doses: The evaluation of the trial’s findings is complicated by the absence of information regarding the mean effective or tolerated doses of the medications. Lastly, the cohort’s investigation of primary aldosteronism as a potential cause of resistant HT is unclear, which introduces a substantial bias. This oversight is crucial because spironolactone is frequently the preferable treatment, and undiagnosed primary aldosteronism is present in over 10% of resistant HT cases. The results may have been distorted by the absence of consideration for this condition, particularly in relation to the observed superior response to spironolactone in comparative studies to other treatments.

Nevertheless, even if spironolactone is the first choice as add-on therapy in RHT, its use is limited by the frequent adverse effects. A retrospective evaluation of 344 patients from the HT Clinic at Odense University Hospital revealed a 4.1% discontinuation due to hyperkalemia, 18% experiencing adverse effects, and lead to a 9.9% discontinuation. Additionally, 5.2% of male patients developed gynecomastia [30]. Therefore, HT management clinical guidelines recommend doxazosin as add-on therapy for patients with RHT who develop adverse effects [8, 9]. Furthermore, in clinical settings where frequent monitoring of serum electrolytes is challenging, doxazosin may be a particularly safer option compared to spironolactone, given the risk of unrecognized hyperkalemia associated with mineralocorticoid receptor antagonists.

(2) Additional benefits of Doxazosin.

In controlled clinical trials, doxazosin has been shown to decrease the levels of total cholesterol, low-density lipoprotein (LDL) cholesterol, and triglycerides, while simultaneously increasing the levels of high-density lipoprotein cholesterol. Doxazosin seems to increase LDL receptor activity, decrease intracellular LDL synthesis, stimulate lipoprotein lipase activity, and reduce the synthesis and secretion of very low-density lipoprotein cholesterol, all of which have direct and indirect effects on lipid metabolism. Platelet aggregation may also be inhibited by doxazosin [31]. The Treatment of Mild Hypertension Study (TOMHS) was a multicentre, randomized, double-blind, parallel-group clinical trial conducted to compare long-term plasma lipid changes among six antihypertensive treatment interventions for stage I (mild) hypertension. The study involved 902 men and women aged 45 to 69 years, with stage I diastolic hypertension. Participants were randomized to one of six treatment groups: placebo, beta-blocker (acebutolol), CCB (amlodipine), diuretic (chlorthalidone), α1-adrenergic blocker (doxazosin), and ACE inhibitor (enalapril). Decreases in plasma total cholesterol and LDL cholesterol were greater with doxazosin and acebutolol, less with chlorthalidone and placebo. Decreases in triglycerides were greater with doxazosin and enalapril, least with acebutolol. Increases in HDL cholesterol was greater with enalapril and doxazosin, least with acebutolol [32].

The Japan Morning Surge1 Study, an open label multicentre trial, enrolled 611 hypertensive patients with self-measured morning systolic BP levels over 135 mmHg while taking antihypertensive drugs. The patients were randomly allocated to an experimental group, receiving bedtime administration of 1 to 4 mg doxazosin, or a control group without any add on medication. The urinary albumin/creatinine ratio was investigated at baseline and 6 months after randomization. Results showed that both morning and evening BP and urinary albumin/creatinine ratio were significantly reduced in the doxazosin group compared to the control group. This difference was more significant in patients with microalbuminuria. The reduction of urinary albumin/creatinine ratio was significantly associated with the use of doxazosin and the change in all self measured BP, and these associations were independent of each other. In conclusion, adding a bedtime dose of doxazosin significantly reduced BP and urinary albumin excretion rate, particularly in those with microalbuminuria [33].

Excessive morning BP surge and orthostatic hypertension, associated with each other, are the risk of cardiovascuar disease [34, 35]. These hypersympathetic phyenotypes of hypertension, are possible responders of renal denervation [36], and are supressed partly by the doxazosin [37].

The dual treatment effect of α1-adrenergic blockers on both elevated BP and lower urinary tract symptoms makes benign prostatic hyperplasia a compelling indication. α1-blockers would be necessary as an adjunctive therapy for a significant number of patients with RHT. Only long-acting α1-adrenergic blockers, like doxazosin-ER-GITS 4–8 mg daily, should be selected from the currently available agents [33].

## Doxazosin ER-GITS and the risk of heart failure

The ALLHAT trial demonstrated that doxazosin was equally effective as chlorthalidone in preventing fatal coronary heart disease or nonfatal myocardial infarction in high-risk hypertensive patients. But doxazosin immediate release was linked to a much higher risk of CHF, which was a secondary goal of the trial. The heightened CHF risk was uniform across diverse subgroups and could not be entirely elucidated by negligible variations in blood pressure. Alpha-blockers may raise the risk of heart failure by raising the levels of norepinephrine and plasma volume. Nonetheless, there may have been a confounding effect resulting from the cessation of previous diuretic therapy [38].

However, in the ASCOT trial, which used extended-release doxazosin (GITS) as a third-line treatment, on the other hand, showed no higher risk of heart failure but better BP and lipid profiles. This indicates that the elevated CHF risk noted in ALLHAT may have been affected by the administration of short-acting doxazosin and additional factors.

Clinical implications suggest that alpha-blockers, particularly doxazosin GITS, can be safely employed as adjunctive therapy for uncontrolled HT without elevating the risk of congestive heart failure (CHF). Present guidelines advocate their application chiefly for benign prostatic hyperplasia and as a fourth-line antihypertensive alternative. Recent findings generally endorse the revaluation of alpha-blockers as safe and effective for HT management, challenging the adverse perception stemming from the ALLHAT trial [39].

## Summary

### Major clinical studies on doxazosin

Doxazosin demonstrated effectiveness in resistant HT (PATHWAY-2), improved lipid profiles (TOMHS), and reduced morning BP surges and urinary albumin/creatinine ratios (Japan Morning Surge-1), with added benefits in lipid metabolism and platelet inhibition, supporting its use in resistant HT and benign prostate hypertrophy.

### Key findings

HT affects over 1.2 billion people worldwide, with BP control remaining poor, particularly in low- and middle-income regions. Stricter BP targets (<130/80 mmHg) are recommended, but challenges like cost, side effects, and nonadherence limit success. Doxazosin-ER-GITS, a controlled-release α1-adrenergic blocker, provides stable BP control with fewer side effects and may be a valuable option for RHT patients, especially those who cannot tolerate spironolactone [40].

### Future directions or implications for clinical practice, literature gap

The article highlights challenges in achieving optimal BP control, particularly for high-risk groups like the elderly, diabetics, and those with chronic kidney disease. It stresses the need for personalized treatment, especially for RHT, which requires more specialized care. Doxazosin, particularly ER-GITS formulations, may offer benefits in improving adherence and reducing side effects. It is particularly useful for elderly patients and those with comorbidities, offering both cardiovascular and lipid benefits. In low-resource settings, affordable combination therapies could improve BP control. However, long-term studies on doxazosin-ER-GITS are lacking, and more research is needed to explore its role in complex HT cases. Global disparities in HT control and the role of non-pharmacological interventions also require attention. Improving diagnosis and monitoring techniques could enhance overall HT management.

As for the concerns of using Doxazosin ER-GITS in low- and medium-income patients, in a cost-effectiveness analysis comparing doxazosin with beta-blockers (atenolol), doxazosin was found to be more cost-effective despite having up to a 30% higher unit price. This advantage is attributed to its favorable effects on lipid profiles and broader clinical outcomes [41]. Doxazosin has also demonstrated cost-effectiveness when used as part of a combination regimen in hypertensive patients with type 2 diabetes in both the UK and Italy [42].
